# Application of machine learning algorithms for accurate determination of bilirubin level on in vitro engineered tissue phantom images

**DOI:** 10.1038/s41598-024-56319-4

**Published:** 2024-03-12

**Authors:** Yijia Yuan, Jiayao Huang, Jiachen Yu, Justin Kok Soon Tan, Kevin Ziyang Chng, Jiun Lee, Sangho Kim

**Affiliations:** 1 Advanced Innovation in Micro/Nanoengineering (AIM) Laboratory, Department of Biomedical Engineering, National University of Singapore, Singapore, 119276 Singapore; 2 N.1 Institute for Health, National University of Singapore, Singapore, 119276 Singapore; 3 College of Biomedical Engineering and Instrument, Zhejiang University, Hangzhou, 310027 China; 4 AI Singapore, Singapore, 117602 Singapore; 5 Department of Neonatology, National University Health System, Singapore, 119228 Singapore; 6 Department of Paediatrics, National University of Singapore, Singapore, 119228 Singapore

**Keywords:** Neonatology, Health care, Medical research, Engineering, Biomedical engineering, Mathematics and computing, Computer science

## Abstract

Neonatal Jaundice is a common occurrence in neonates. High excess bilirubin would lead to hyperbilirubinemia, leading to irreversible adverse damage such as kernicterus. Therefore, it is necessary and important to monitor neonates’ bilirubin levels in real-time for immediate intervention. However, current screening protocols have their inherent limitations, necessitating more convenient measurements. In this proof-of-concept study, we evaluated the feasibility of using machine learning for the screening of hyperbilirubinemia in neonates from smartphone-acquired photographs. Different machine learning models were compared and evaluated to gain a better understanding of feature selection and model performance in bilirubin determination. An in vitro study was conducted with a bilirubin-containing tissue phantom to identify potential biological and environmental confounding factors. The findings of this study present a systematic characterization of the confounding effect of various factors through separate parametric tests. These tests uncover potential techniques in image pre-processing, highlighting important biological features (light scattering property and skin thickness) and external features (ISO, lighting conditions and white balance), which together contribute to robust model approaches for accurately determining bilirubin concentrations. By obtaining an accuracy of 0.848 in classification and 0.812 in regression, these findings indicate strong potential in aiding in the design of clinical studies using patient-derived images.

## Introduction

Neonatal jaundice or hyperbilirubinemia is one of the most common clinical conditions in the neonatal period, affecting 60% of term and 80% of preterm babies in their first week of life^1^. As compared to adults, most babies are born with a developmentally immature liver system and neonatal haemoglobin has a relatively shorter lifespan, resulting in a less effective enterohepatic circulation process and a much slower rate of bilirubin excretion that is unable to keep up with its production. Congenital diseases such as glucose-6-phosphate dehydrogenase (G6PD) deficiency can also cause the abnormality of bilirubin excretion^2^. The results in the accumulation of the non-excreted bilirubin in the sclera or subcutaneous tissue, manifesting in their characteristic yellow colouration^3,4^. Although in most babies, physiological jaundice would show up during the first week of birth and gradually disappear after two weeks as the neonatal liver system develops, there are cases that the bilirubin level increases too fast, or it becomes very high. Circulating unconjugated bilirubin can cross the neonatal blood–brain barrier, destroying the brain cells in a process known as kernicterus, which is the most severe form of neurotoxicity in neonatal jaundice^5^. If hyperbilirubinemia is not treated in time, neonates could be less active and develop seizures, resulting in deafness, cerebral palsy, mental retardation, irreversible brain damage, or even death^6,7^. Although many jaundice conditions can be effectively managed with phototherapy^8^, or in severe cases, exchange transfusions^9^, timely detection of hyperbilirubinemia in neonates is still critical in preventing adverse sequelae.

The measurement of total serum bilirubin (TSB) remains the gold standard for diagnosing and monitoring jaundice in neonates^10^. However, TSB measurements require invasive heel-prick sampling that is not only time-consuming and limited to clinical use but can also lead to local infection or even anaemia in cases of frequent sampling^3,11–13^. Furthermore, the procedure can be distressing to both the newborns and their parents^14,15^. In lieu of this, visual examination can detect jaundice when bilirubin levels are higher than 85 μmol/L^16^. However, it cannot estimate the concentration of bilirubin itself. Consequently, low accuracy and high variation in the visual assessment results have been reported in the past, demonstrating its low reliability^3^. Another alternative is transcutaneous bilirubinometry (TcB), a non-invasive method that provides an instantaneous readout of the skin bilirubin level based on spectroscopic measurements^3,13^. Although widely used in hospitals to guide jaundice management, it is known to be susceptible to interferences from skin pigmentation and presents wide limits of agreement with the TSB values^17,18^. Furthermore, the high cost of transcutaneous bilirubinometers precludes its use for home-based jaundice screening. These illustrate the constraints of hyperbilirubinemia screening, especially when there is a need for routine hyperbilirubinemia monitoring after hospital discharge^19,20^. Meanwhile, it is notable that diagnostic errors contribute to about 10% of patient mortality and up to 17% of patient complications and arise out of misjudgement or overlooking of relevant information^21^. In view of this constraint, there is an increasing presence of machine learning (ML) and neural networks in the field of medical diagnostics research. A well-trained ML algorithm obtained through robust supervised learning with big data sets can reduce the incidence of such errors by identifying more definitive information clusters. Indeed, ML has been employed for jaundice monitoring. One of such examples is BiliCam, a smartphone application which estimates bilirubin levels from images of neonates’ sternums^22^. The use of a colour calibration card allows for colour balancing to eliminate variations in the environmental illumination. A modest performance was reported when compared with the TSB readings (linear correlation of 0.84 with a mean error of 2.0 mg/dl), and the authors had discussed several limitations in their study, such as the difficulty in image segmentation and the neglect of the confounding effect of skin tone. Furthermore, the use of a cyan-magenta-yellow(-key) (CMY(K)) colour calibration card has a limited representation of the wide range of natural colours^23^. Such colour calibration cards require proper usage, storage, and cleaning to prevent the folding, dusting, and greasing of the card, which can lead to potentially inaccurate prediction of the bilirubin level^23^.

Moreover, the possible confounding effects of biological factors such as measurement site, skin light scattering property, skin tone, as well as external environmental factors, such as lighting and camera parameters, have not been characterized. The development of skin thickness is also a part of the maturing process of neonates and differs around the body. Previous research has shown a positive correlation between the epidermal thickness and the pigmentation of the skin, suggesting that the measurement site may demonstrate different bilirubin levels in screening^24–26^. Additionally, the natural occurrence of variation in the light scattering property of the skin may not only affect the conventional screening methods, such as TcB measurement but also cause the variation of skin features in neonates to be varied^27,28^. Other than biological factors, camera parameters such as ISO and white balance, and environmental factors such as lighting conditions may cause the variation of the photo collected in mobile phone screening.

In this study, we used an in vitro tissue phantom to systematically evaluate the confounding effects of the aforementioned biological, environmental and hardware factors on the determination of bilirubin levels. Supervised ML models were developed for classification and regression analyses of photographs of the tissue phantom containing varying concentrations of bilirubin. Various image preprocessing and colour mapping strategies were investigated for their efficacy in improving the ML model performance.

## Results

### Colour space channel sensitivity analysis

**Figure 1:**
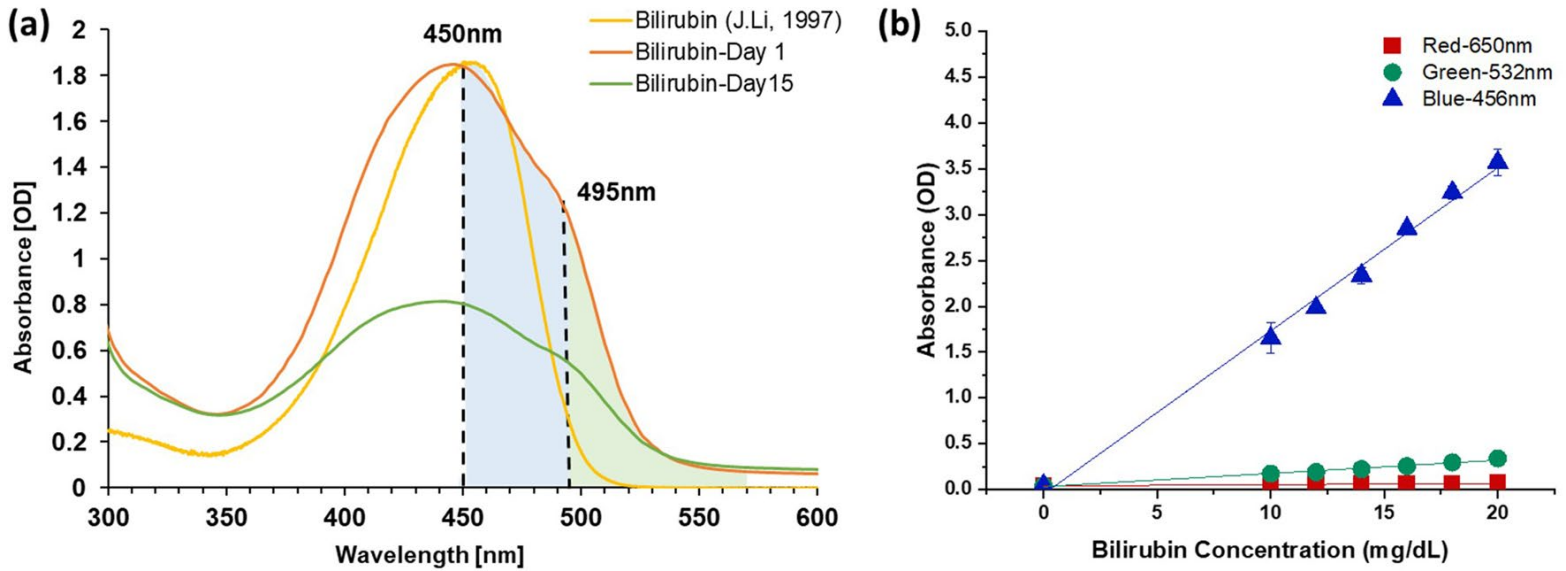
Spectral characterization of bilirubin solutions with different concentrations. (**a**) Spectral characterization of bilirubin solution at different days. Red curve represents the freshly made bilirubin. A peak shift towards left has been observed for stored bilirubin due to conversion of bilirubin to biliverdin. (**b**) Scatter plot of absorbance of bilirubin solution in different colour wavelengths; Statistical differences (P < 0.01) in both correlation and sensitivity were observed among all three wavelengths.

An absorbance spectral scan was performed on freshly prepared bilirubin samples using a microplate reader and compared against reported spectral profiles to ensure the absence of biliverdin. The solutions were shown to strongly absorb light in the blue colour region (Fig. 1a), with an absorbance peak at 450nm^29^. We then sought to confirm the spectral behaviour of our prepared bilirubin samples by verifying the linearity of the optical density (OD) at three wavelengths—red (R, 650 nm), green (G, 532 nm) and blue (B, 456 nm)—with varying bilirubin concentrations. These three wavelengths also correspond to the RGB colour filters of the mobile phone digital camera sensor^30^. As shown in Fig. 1b, a strong linear correlation (R^2^ = 0.996) and relatively higher sensitivity (slope = 0.198) were observed in the blue wavelength, indicating that the blue wavelength has demonstrated the strongest bilirubin signal and it is more sensitive to the changes in the bilirubin concentration. Similarly, the green wavelength also displayed a strong correlation (R^2^ = 0.983), but a much lower sensitivity (slope = 0.017) as compared to the blue wavelength. The red wavelength had the lowest correlation (R^2^ = 0.735) and sensitivity (slope = 0.002) among all three wavelengths, suggesting the red channel might not respond sensitively to the changes in the bilirubin concentration.

### Confounding effects of biological and external factors

**Figure 2:**
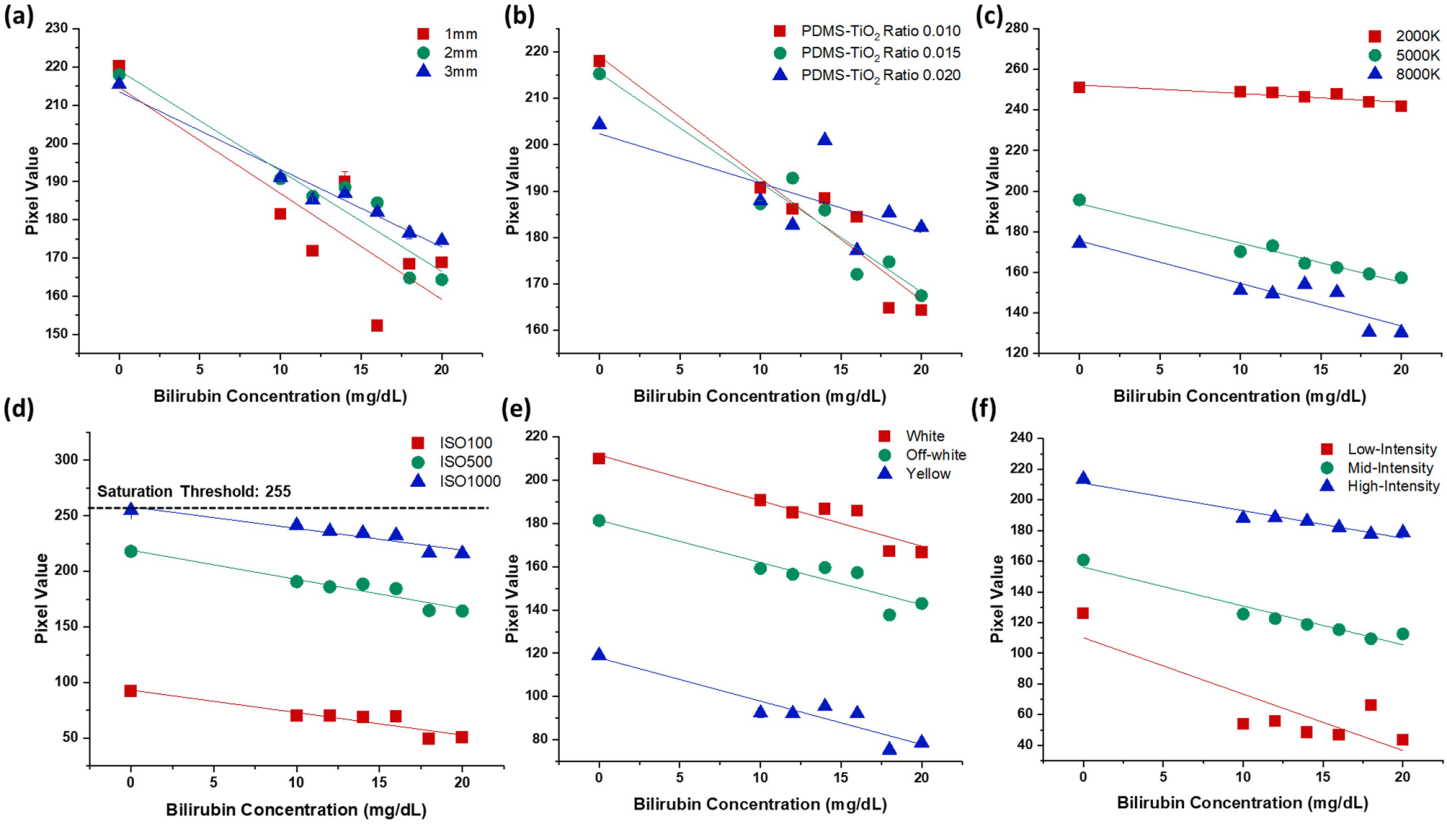
Parametric study of important biological and external features. (**a**) Scatter plot of the pixel value of bilirubin concentrations in PMDS-TiO_2_ tissue phantom samples with different thicknesses; A statistically significant difference (P < 0.05 in 1 mm, P < 0.001 in 2 mm and P < 0.001 in 3 mm) was observed in the sensitivity slope but not in correlation among different thicknesses. (**b**) Scatter plot of the pixel value of bilirubin concentrations in samples with different light scattering ratios (PDMS to TiO_2_ ratio); Significant statistical difference was observed in both correlation and sensitivity between 0.01 and 0.02 PDMS-TiO_2_ ratio (P < 0.005), as well as between 0.015 and 0.02 PDMS-TiO2 ratio (P < 0.005). (**c**) Scatter plot of the pixel value of bilirubin concentrations in samples with different WB. Significant statistical difference was observed in both correlation and sensitivity among 2000 K (P < 0.05), 5000 K (P < 0.005) and 8000 K (P < 0.01). (**d**) Scatter plot of the pixel value of bilirubin concentrations in samples with different ISOs; ISO200 and ISO500 (P < 0.05), ISO200 and ISO700 (P < 0.05), as well as ISO500 and ISO700 (P < 0.05) datasets are statistically different from each other in correlation. At the 0.05 significance level, a significant statistical difference was also observed in sensitivity between the ISO200 and ISO500 datasets (P < 0.05), as well as between the ISO200 and ISO700 datasets (P < 0.05). (**e**) Scatter plot of the pixel value of bilirubin concentrations in samples with different illumination tones; A significant statistical difference was observed in correlation among all channels (P < 0.05). (**f**) Scatter plot of the pixel value of bilirubin concentrations in sample images with different light intensities; Different light intensities have demonstrated a considerable statistical significance in both sensitivity and correlation (P < 0.05).

In vitro evaluation and characterization of the ML-based bilirubin measurement were conducted using an elastomeric tissue phantom mimicking neonatal skin with varying concentrations of bilirubin. Parametric studies of the various biological and external factors were conducted to evaluate the isolated effect of each biological and external factor through tight control of the in vitro fabrication parameters and environmental conditions. First, Polydimethylsiloxane-Titanium dioxide (PDMS-TiO_2_) tissue phantom samples with varying thicknesses were evaluated (Fig. 2a). Relatively strong correlations between blue channel pixel value and different bilirubin concentrations were observed in all 1 mm (R^2^ = 0.722, slope = −2.775), 2 mm (R^2^ = 0.921, slope = −2.630) and 3 mm (R^2^ = 0.972, slope = −2.033) samples. However, a statistically significant difference (P < 0.05) in sensitivity was observed between 1 mm samples and 3 mm samples, as well as between 2 mm samples and 3 mm samples, demonstrating that the images from the 3 mm samples were less responsive to the changes in the bilirubin concentration.

Next, images of PDMS-TiO_2_ tissue phantom samples with different extents of light scattering (varying ratios of PDMS to TiO_2_ light scattering agent) were used to represent skin containing varying amounts of scattering agents such as collagen and adipose tissue^31^. As shown in Fig. 2b, strong correlations between blue channel pixel values and different bilirubin concentrations were observed in samples with a ratio of 0.01 (R^2^ = 0.921, slope = −2.630) and samples with a ratio of 0.015 (R^2^ = 0.936, slope = −2.359) respectively. However, no statistically significant linear regression relationship (R^2^ = 0.482, slope = −1.065, P > 0.05) was observed between blue channel pixel values and different bilirubin concentrations in PDMS-TiO_2_ tissue phantom images with a TiO_2_ ratio of 0.02.

Two hardware parameters that are commonly adjusted for image quality control were evaluated. Firstly, PDMS-TiO_2_ tissue phantom images were captured under different white balance (WB) conditions (Fig. 2c). When WB is much lower (2000 K) than the actual colour temperatures, weaker correlation and sensitivity (R^2^ = 0.774 slope = −0.425) were observed, suggesting a strong confounding effect on the bilirubin concentration prediction. However, as the WB approached 5000 K, which is close to the actual colour temperature in the image collection environment, a much stronger correlation and sensitivity (R^2^ = 0.963, slope = –1.940) were observed in the scatter plot. As the WB increased higher (8000 K), while the sensitivity remained unchanged (slope = −2.097, P > 0.05), a weaker correlation (R^2^ = 0.848) was observed. Secondly, camera sensor light sensitivity (ISO) was varied to establish its impact on the bilirubin estimation. Despite yielding moderately high sensitivities, it was observed that at low and high ISO values, a relatively weaker correlation was observed (ISO100, R^2^ = 0.898, slope = −1.944; ISO1000, R^2^ = 0.868, slope = −2.022) compared to the medium ISO (ISO500, R^2^ = 0.921, slope = −2.630) in response to the changes in bilirubin concentrations. As expected in Fig. 2d, the pixel intensity values increased with increasing ISO values. By extrapolation, it is expected that the pixel value of the diluted bilirubin can be saturated under brighter or darker lighting conditions. This suggests that an appropriate ISO setting needs to be selected to control the image brightness and prevent signal saturation at the lower and higher bilirubin.

The effect of ambient lighting conditions (light intensity and illumination tone) was also investigated (Fig. 2e). Statistically significant differences in the correlation and sensitivities were calculated between the low, moderate and high light intensity conditions. It was observed that images with low light intensity generated a weak correlation (R^2^ = 0.717) but high sensitivity (slope = −3.678). The correlation was observed to increase when the light intensity increased from moderate (R^2^ = 0.934) to high intensity (R^2^ = 0.942) while the sensitivity slope decreased from −2.531 to −1.793. These results suggest that high but appropriate amount of light intensity would aid the camera pixels in response to collect the colour information from the PDMS-TiO_2_ tissue phantom images.

Lastly, different illumination tones were supplied and tested using a diffused light source (Fig. 2f). A relatively strong linear correlation was observed in all three illumination tones—white light (R^2^ = 0.894, slope = −2.106), off-white light (R^2^ = 0.860, slope = −1.953) and yellow light (R^2^ = 0.878, slope = −2.000). All three conditions demonstrated satisfactory (~ 0.9) correlation and sensitivity in response to the changes of bilirubin concentrations in the PDMS-TiO_2_ tissue phantom images. However, as compared to the pixel value in the images with white light, it is observed that the pixel values are generally lower in images with off-white light and are much lower in images with yellow light. This is expected as the spectral characterization (see Supplementary Fig. 1) of illumination tones demonstrated the strongest blue light scattering signal (~ 450 nm) in white light, followed by off-white light and yellow light.

The last factor tested (capture distance away from the subject) did not demonstrate any statistical significance in correlation and sensitivity (P > 0.05), suggesting that distance does not affect the image information of the PDMS-TiO_2_ tissue phantom images (see Supplementary Fig. 2).

### Angular error in different white balance (WB) corrections

We have shown that the bilirubin level prediction from images is strongly dependent on the colour representation. This necessitates the correction of the WB to achieve higher colour rendering. We applied different colour constancy algorithms (GW, MSGP, MaxRGB, and CH) and compared the WB-corrected images against the corresponding ground truth images captured with a light temperature of 5600 K. The relative performances of the various WB correction methods are shown in Table 1. The different WB correction methods produce considerable differences, and the angular error results showed that the Gray World (GW) method performed the best at 3000 K (mean angular error: 0.44) for the PDMS-TiO_2_ tissue phantom images. We also conducted additional tests to assess the GW accuracy in correcting raw images captured at different colour temperatures (Table 2). The results indicated that the GW method consistently produced a low angular error (< 0.5) for all groups, demonstrating the stability and effectiveness of the GW method in correcting the WB variations of tissue phantom images.

**Table 1:** Comparison of mean angular errors and standard deviation of different white balance methods for 3000 K WB PDMS-TiO_2_ tissue phantom images under single light (colour temperature: 5600 K).

		MSGP			WP		GW		CH	
	Mean		SD	Mean		SD	Mean	SD	Mean	SD
3000 K	4.22		1.51	1.62		0.37	0.44	0.23	0.48	0.25

*MSGP* mean shift grey pixel, *GW* gray world, *WP* white patch, *CH* Cheng’s PCA, *GT* ground truth.

**Table 2:** Comparison of mean angular errors and standard deviation of gray world for 3000 K to 7000 K WB PDMS-TiO_2_ tissue phantom images under single light (colour temperature: 5600 K).

2000 K		3000 K		4000 K		5000 K		6000 k				7000 K	
Mean	SD	Mean	SD	Mean	SD	Mean	SD	Mean		SD		Mean	SD
0.43	0.10	0.44	0.23	0.33	0.08	0.38	0.07	0.30	0.14		0.33		0.17

### Colour spaces conversion

**Figure 3:**
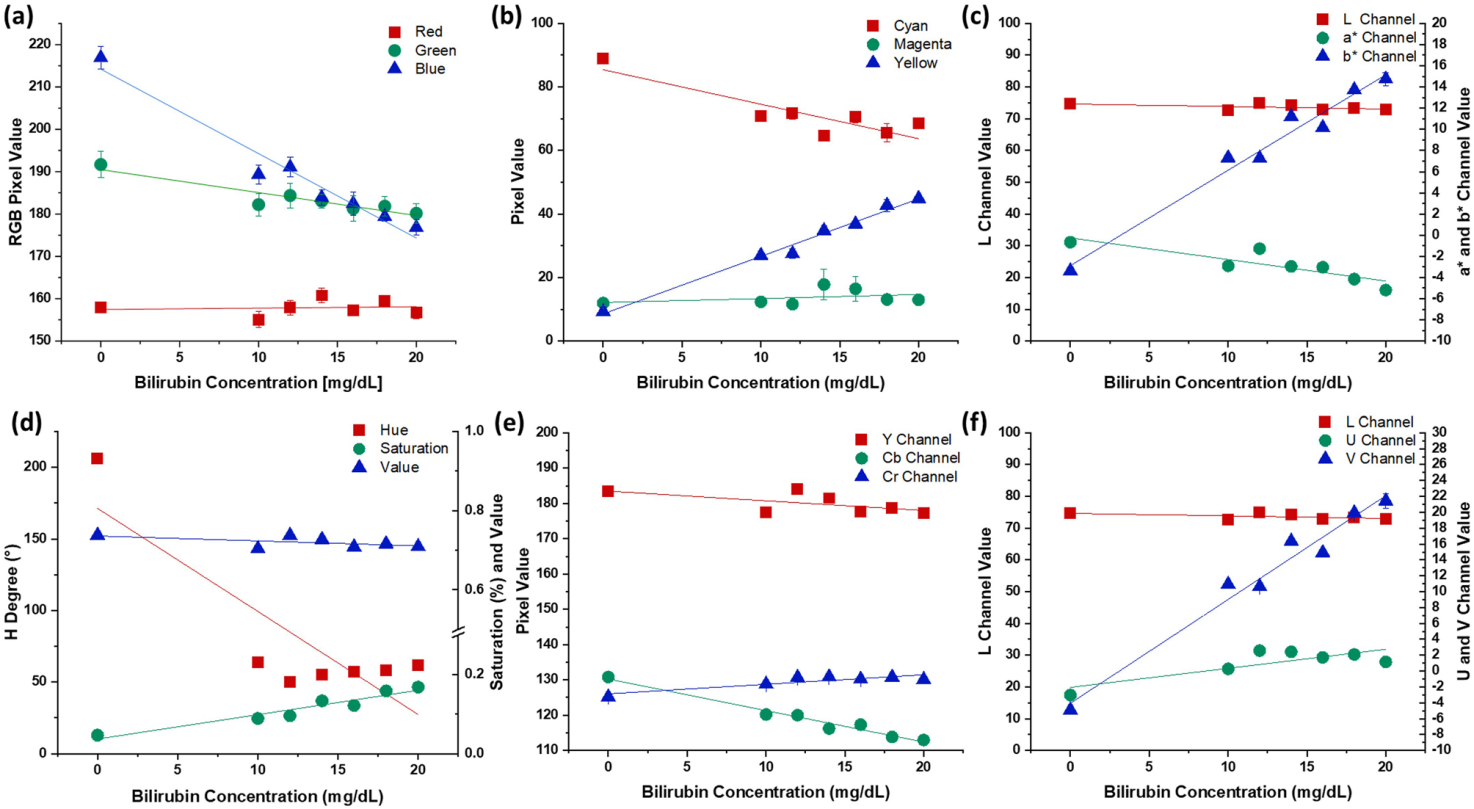
Linear regression and evaluation of important feature channels from colour spaces—RGB, CMYK, L*a*b*, HSV, YCbCr and LUV. (**a**) Scatter plot of pixel values of bilirubin concentrations of PDMS-TiO_2_ tissue phantom samples in the RGB channels respectively; Significant statistical difference in correlation was observed between the B and R channel (P < 0.05), as well as between the B and G channel (P < 0.005). (**b**) Scatter plot of pixel values of bilirubin concentrations of samples in the CMY(K) channels respectively; A significant statistical difference in both correlation and sensitivity was observed among all channels (P < 0.05). (**c**) Scatter plot of pixel values of bilirubin concentrations of samples in the CIELAB channels respectively. A statistically significant correlation (P < 0.05) was observed in correlation among all channels. Sensitivity also demonstrated a significant statistical difference between the L channel and the a* channel, as well as between the L channel and the b* channel (P < 0.05). (**d**) Scatter plot of pixel values of bilirubin concentrations of samples in the HSV channels respectively. A significant statistical difference in both sensitivity and correlation was observed among all channels (P < 0.05). (**e**) Scatter plot of pixel values of bilirubin concentrations of samples in the YCbCr channels respectively. A significant statistical difference (P < 0.05) was observed in both correlation and sensitivity among all different channels. (**f**) Scatter plot of pixel values of bilirubin concentrations of samples in the LUV channels respectively. A significant statistical difference (P < 0.05) was observed in both correlation and sensitivity among all different channels.

Similar to the WB correction, we evaluated several colour spaces for their correlation with the bilirubin concentration using the tissue phantom images taken in a controlled image collection environment. As shown in Fig. 3a, like the spectrophotometric characterization presented in Fig. 1a, a strong linear correlation and high sensitivity (R^2^ = 0.958, slope = −1.995) were observed between the blue channel pixel values and bilirubin concentrations in PDMS-TiO_2_ tissue phantom images. A relatively lower correlation and sensitivity were observed in the green channel pixel value (R^2^ = 0.871, slope = −0.542), corresponding to a lower accuracy and sensitivity as compared to the blue channel. The pixel value acquired from the red channel did not demonstrate a statistically significant linear relationship (R^2^ = 0.014, slope = 0.034, P < 0.05) with the bilirubin solutions, suggesting an insensitive response to the changes in bilirubin concentration.

Mapping of the RGB values to the CMY(K) colour space was also evaluated (Fig. 3b). Among all channels, the pixel values obtained from the Y channel were observed to show the strongest linear correlation (R^2^ = 0.986) and the highest sensitivity (slope = 1.812) whereas the C channel showed a weaker correlation (R^2^ = 0.782) and a less sensitive response (slope = −1.086) to the changes in bilirubin concentrations. The M channel showed the weakest correlation (R^2^ = 0.122) with bilirubin level.

In the CIELAB (L*a*b*) colour space (Fig. 3c), values acquired from the b* channel displayed a strong linear correlation (R^2^ = 0.971) and relatively high sensitivity (slope = 0.902) to the changes in bilirubin concentrations. The value in the a* channel had a comparably weaker correlation (R^2^ = 0.749) and a lower sensitivity (slope = −0.203). Notably, the pixel value obtained from the L channel did not show a statistically significant linear relationship (R^2^ = 0.3019, slope = −0.08, P > 0.05) with varying bilirubin concentrations.

Meanwhile, Fig. 3d indicated the linear relationship between bilirubin level and the channels in Hue-Saturation-Value (HSV) colour space respectively. In the S channel, a strong linear correlation, which an R^2^ value is 0.917, was observed between the bilirubin concentration and the S channel value. Sensitivity was also observed to be relatively strong (slope = 0.0061) as compared to other channels in the HSV colour space, showing strong capability in response to the change of bilirubin concentrations.

The YCbCr channel colour space demonstrated a relatively linear relationship between the 3 channels and the bilirubin concentration, albeit with an evidently lower sensitivity (Fig. 3e). A very strong linear correlation and moderate sensitivity were observed in the Cb channel with an R^2^ value of 0.970 and a slope of −0.892. Compared to the other channels which demonstrated relatively less strong linear correlation and sensitivity (Y Channel: R^2^ = 0.769, slope = 0.270; Cr Channel: R^2^ = 0.381, slope = −0.275), the Cb channel displayed superior sensitivity to changes in bilirubin concentration.

The final colour space mapping evaluated was the CIELUV (L*u*v*) colour space (Fig. 3f). As compared to the L (slope = −0.080) and u* (slope = 0.240) channels, which have either low or statistically insignificant sensitivity (P > 0.05) to the changes of bilirubin value, the v* channel does not only have a strong linear correlation (R^2^ = 0.969), but also a relatively higher sensitivity (slope = 1.308) to changes in bilirubin concentration.

### Classification model performance

**Figure 4:**
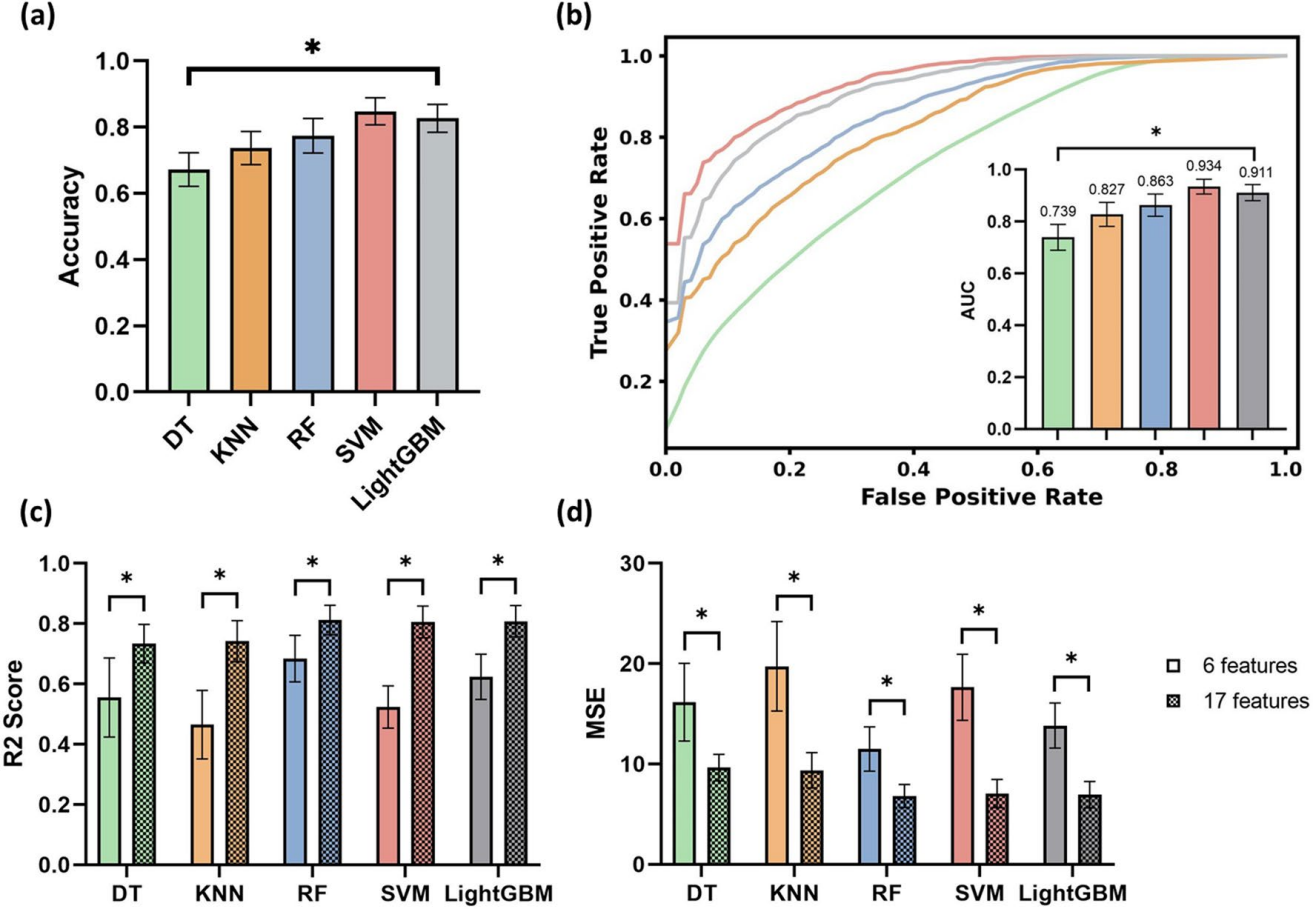
Model performances. (**a**) Accuracy performance among DT, KNN, RF, SVM and LightGBM models in the classification task; A significant statistical difference in accuracy was observed among models (P < 0.05). All models demonstrated a significant statistical difference in pairwise comparison except for the comparison between SVM and LightGBM model (P > 0.05). (**b**) ROC performance of the five different models. The inlet graph shows the AUC performance, which represents the capability of the model to distinguish between the tissue phantom images with normal bilirubin levels and those images with abnormal bilirubin concentrations. The AUC demonstrated a significant statistical difference among all models (P < 0.05). (**c**) R^2^ value among different models in the regression task with a different number of features as training labels. A significant statistical difference (P < 0.05) was observed in R^2^ between 6 and 17 features among all models. Five models are statistically different from each other except the RF, SVM and LightGBM in pairwise comparison. (**d**) MSE value among different models in the regression task with a different number of features as training labels. A significant statistical difference (P < 0.05) was observed in MSE between 6 and 17 features among all models. Asterisk (*) indicates P < 0.05.

Five machine learning models—decision tree (DT), K-nearest neighbour (KNN), random forest (RF), support vector machine (SVM), and LightGBM—were evaluated for their accuracy in performing binary classification of jaundice based on the PDMS-TiO2 tissue phantom image data (Fig. 4a). The mean accuracy was 0.672 in DT, 0.737 in KNN, 0.774 in RF, 0.827 in LightGBM and 0.848 in SVM. Statistically significant differences (P < 0.05) in accuracy were also observed among the models. The pairwise comparison also showed that among all models tested, SVM performed the best in the bilirubin binary classification task, followed by the LightGBM, RF and KNN, with DT performing the worst. The corresponding receiver-operating-characteristic (ROC) curves and the respective area-under-curve (AUC) scores further validated this observation (Fig. 4b). The AUC scores obtained for each model were as follows: 0.74 (DT), 0.82 (KNN), 0.86 (RF), 0.91 (LightGBM), and 0.93 (SVM). The results were consistent with the performance comparison based on the cross-validated accuracy, suggesting that the SVM model has the best predictive capability among all other models tested.

In addition, as shown in Fig. 4c, d, we also tested the model capability in performing the regression task, evaluating the performance of each regression model (DT, RF, KNN, SVR, and LightGBM) in predicting the exact bilirubin concentration in the PDMS-TiO_2_ tissue phantoms. When the models were trained with limited features, a relatively large mean square error, MSE, was observed in all models (DT: 16.157; KNN: 19.749; RF: 11.499; SVM: 17.651 and LightGBM: 13.830). A low R^2^ score (DT: 0.555; KNN: 0.465; RF: 0.684; SVM: 0.524 and LightGBM: 0.624) was observed at the same time. However, the results showed significant improvements (P < 0.05) in the model performances (R^2^ score) when additional features were included for all five models (DT: 0.734; KNN: 0.742; RF: 0.812; SVM: 0.806 and LightGBM: 0.808). The corresponding MSE was also greatly reduced across all models (DT: 9.635; KNN: 9.366; RF: 6.816; SVM: 7.054 and LightGBM: 6.946). Similar to the classification task, LightGBM, SVM and RF models performed the best in predicting bilirubin concentrations. The acquired low MSE value, akin to the square of bilirubin level variance, additionally indicates a minor disparity in relation to the true bilirubin levels. A variance ranging from ± 2.61 mg/dl to ± 3.10 mg/dl was achieved across all evaluated models, thus illustrating the predictive capacity of machine learning models in determining precise bilirubin levels.

## Discussion

In this study, we systematically evaluated the feasibility of hyperbilirubinemia detection by ML-based analysis of photographs of neonate skin. We also investigated the impact of various biological, hardware and environmental factors on the predictive performance of the bilirubin concentrations in different machine learning models. PDMS was used as the base material in the in vitro study to mimic human skin due to its distinct advantage of maintaining the same and desired optical property over time^32^. The PDMS-TiO_2_ tissue phantom image collection was conducted in a highly controlled environment. Fresh bilirubin was prepared and utilised in the experiment to prevent the oxidation of the bilirubin to biliverdin. A linear relationship between the reflectance and the molar concentration of the bilirubin solution was expected across the experimental cases^33^. By comparing and evaluating the strength of the correlations and the corresponding sensitivities of the various factors, a more suitable and accurate feature set could be identified for improved model performance.

Absorbance spectral scans of the bilirubin solutions in a microplate reader showed strong bilirubin absorbance spanning a wide range of wavelengths in the green and blue bands. Similarly, both the green and blue channels in the RGB colour space extracted from the tissue phantom photographs have been shown to be potential options for image colorimetric evaluation and feature selection, which is expected considering the linear relationship between absorbance and reflectance^34^. Although the red channel does not directly reflect the changes in bilirubin concentrations, its pixel value provides relevant information on the effect of confounders, improving the overall performance of the machine learning models in both classification and regression tasks.

The human visual ability to correct for the colour effects of light sources is known as colour constancy. Unlike human eyes, a digital RGB camera is sensitive to the change in illumination in the external environment. Therefore, appropriate image pre-processing would rectify the actual colour of the images. We have selected several static WB correction algorithms based on the unique characteristics of PDMS-TiO_2_ tissue phantom images, which have more uniform image characteristics across the dataset. Different algorithms may have various performances in the same case study. The GW algorithm is a classical colour constancy algorithm. The algorithm assumes the average reflectance of a scene with rich colour variation is achromatic, which means that any deviation from the average colour of the grey is caused by the influence of the light source^35^. In our case, the PDMS-TiO_2_ tissue phantom images were captured with a single light source, which is consistent with the mechanism by which the GW algorithm processes the entire image. Therefore, by using the GW algorithm, the improvement in image quality from white balance was visually clear, minimizing the possible noise in the light environment during image collection. Other WB correction algorithms also have their pros and cons based on various assumptions. The Mean-Shifted-Grey-Pixels (MSGP) algorithm employs the grey pixels in the natural scene. It assumes that these grey pixels can help estimate the global illumination of an image. This method requires all true grey pixels to be aligned with the grey-light vector for the calculation of the environmental illumination direction, and is suitable for use under controlled lighting conditions^36^. Similarly, the MaxRGB algorithm is also a relatively fast and common WB correction algorithm based on the white patch assumption, which considers the brightest spot as the white point of the image. MaxRGB then rectifies the effects of mismatched illumination^35^. Another possible WB correction algorithm that we have considered was Cheng’s PCA (CH). It is a method based on the assumption that the gradient of an image is achromatic. This algorithm selects the top and bottom parts of the colour points by calculating the projection of all colour points in the colour gamut onto the mean vector direction. The first PCA vector of the data matrix they form is the estimated direction of illumination^37^. In this study, CH algorithm also shows capable performance in the WB correction in the PDMS-TiO_2_ tissue phantom images. The mean and standard deviation (SD) of the angular error is also shown to be generally reliable criteria to evaluate the performance of a WB correction algorithm on specific images. Therefore, our result analysis has demonstrated the necessity of finding the most suitable WB correction algorithms for the specific image pre-processing task.

Colour spaces are taken into consideration as potential features in this study. Different colour space models have different pros and cons in real applications. One of the major limitations of the RGB colour space is the mixing of brightness value and actual colour in the colour channel. The brightness of the image would greatly affect the pixel value of each channel, generating huge variations in the bilirubin concentration prediction. Therefore, all the channels in the other colour spaces mentioned in this paper were studied and evaluated to understand the possible strength in determining the actual bilirubin levels in the PDMS-TiO_2_ tissue phantom images. HSV is a colour space converted from the RGB colour space to an inverted six-column cone. One advantage of the HSV colour space is that it separates the chrominance information from the brightness channel, making the HSV colour space a good light-independent colour space for image processing and analysis. The L*a*b* colour space is a device-independent colour model. The conversion process was based on the CIEXYZ value transformation from the raw RGB value^38^. Furthermore, the CMY(K) colour space is a subtractive colour space that has a direct representation of the yellow colour. Since bilirubin deposited yellowish pigment on the skin, the CMY(K) colour space is expected to evaluate the yellow colour information in the image. Another commonly used colour space, the L*u*v* colour space, was chosen for this study as it is widely used for information visualization analysis due to its stable saturation performance. It also separates the raw RGB information into luminance and chrominance information based on digital standards. Our results had shown the better-performing fitted curves of colour channels versus bilirubin concentration, providing direct and indirect information in PDMS-TiO_2_ tissue phantom image analysis. The fit results showed that the B value in RGB colour space, the Y channel in CMY(K) colour space, the S channel in HSV colour space, the b* channel in L*a*b* colour space and the v* channel in L*u*v* colour space have great potential to predict changes in bilirubin concentration as compared to other colour channels, providing potentially useful colour information. The sensitivity analysis also suggested that these colour channels exhibit strong discrimination of changes in bilirubin concentration and are capable to exclude skin factor interference environment. Therefore, the overall results obtained from different colour spaces suggest a high potential of accurately estimating the actual bilirubin level with the image pixel values acquired from specific colour channels as features for machine learning models.

In this study, we tested several types of supervised learning methods, including some well-known ensemble model structures. The results showed that the ensemble methods generally produce better performance in determining the bilirubin concentration. This is explainable as ensembles integrate the predictions of several individual supervised learning models with a learning algorithm, improving the overall robustness of the exact bilirubin concentration prediction^39^. Furthermore, SVM also demonstrated its high potential and effectiveness in accurately predicting the bilirubin concentration. Based on the results of this study, it is expected that a better model prediction performance could be achieved by integrating the SVM model with the appropriate ensemble model framework. Multiple approaches could be considered to determine the base learner and input features.

**Figure 5:**
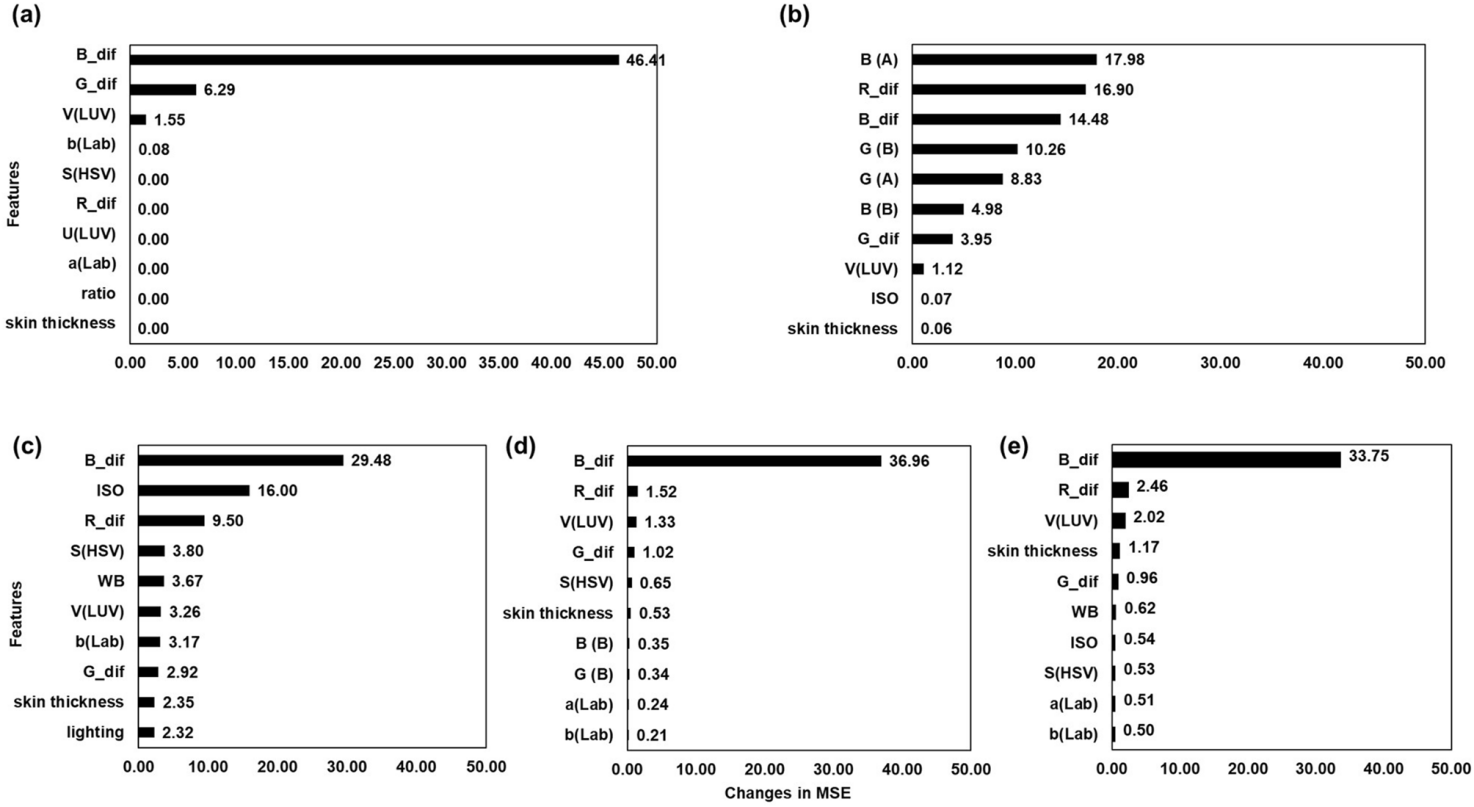
Top 10 important features in different models. (**a**) Top 10 features in the DT model. The difference in the blue channel pixel value between the two ROI regions outperformed other features in the DT model. (**b**) Top 10 features in the KNN model. The ROI blue channel pixel value demonstrated the highest importance. (**c**) Top 10 features in the SVR model. The difference in the blue channel pixel value also showed the highest importance. (**d**) Top 10 features in the RF. A similar observation of the most determining feature was observed. (**e**) Top 10 features in the LightGBM. The difference in the blue channel pixel value between the two ROI regions was the highest determining factor of the model performance in the LightGBM model.

Permutation importance is a well-established method for evaluating the relative importance of the input features and the corresponding impact on model performance, facilitating the understanding of the underlying relationships between the features and the target variable in feature selection and model optimization^40^. In this study, permutation importance also helps to further validate the conclusions generated from the linear regression analysis. As shown in Fig. 5, important features, including the B channel in the RGB colour space and the B channel pixel value difference between two regions of interest (ROIs), have contributed magnificently to the overall model performance. Especially in the ensemble models such as the SVM, RF and LightGBM, which have high accuracy, the features from various colour spaces have a dominant impact on the changes in MSE value. It is also noticeable that lighting condition is a relatively less important feature in the bilirubin tissue phantom study. This can be explained by the fact that the appropriate white balance correction has reduced the confounding effect of the environmental lighting on the task of determining the exact bilirubin concentration^41^. Therefore, with features from different colour spaces, the model becomes more robust to the changes in the collection environment.

However, one of the limitations of this tissue phantom study is tissue phantom samples could not fully represent the birth age of the neonates. In the actual clinical situations, the risk category of hyperbilirubinemia is determined based on a particular bilirubin level (μmol/L) threshold, which varies differently with different neonate’s gestational age, birth weight, time since birth, and additional neurotoxicity risk factors^42^. Another limitation of our proof-of-concept study might be the omission of clinical factors such as skin tone and haemoglobin as inputs to the ML models. As the concentration of melanin varies between neonates, such a variation would affect the reflectance properties of the skin, leading to a possible underestimation or overestimation of the bilirubin level in neonates^43^. Haemoglobin has also been shown to lead to underestimates of TcB readings^44^ and to interfere with the spectra of reflected light^45^. It is of note that these features should also be taken into consideration in the actual clinical prediction of neonatal jaundice. Findings from this study would be used to design a clinical study to evaluate the model performance with patient images and data.

## Methods

### Bilirubin and Tissue Phantom Preparation

**Figure 6:**
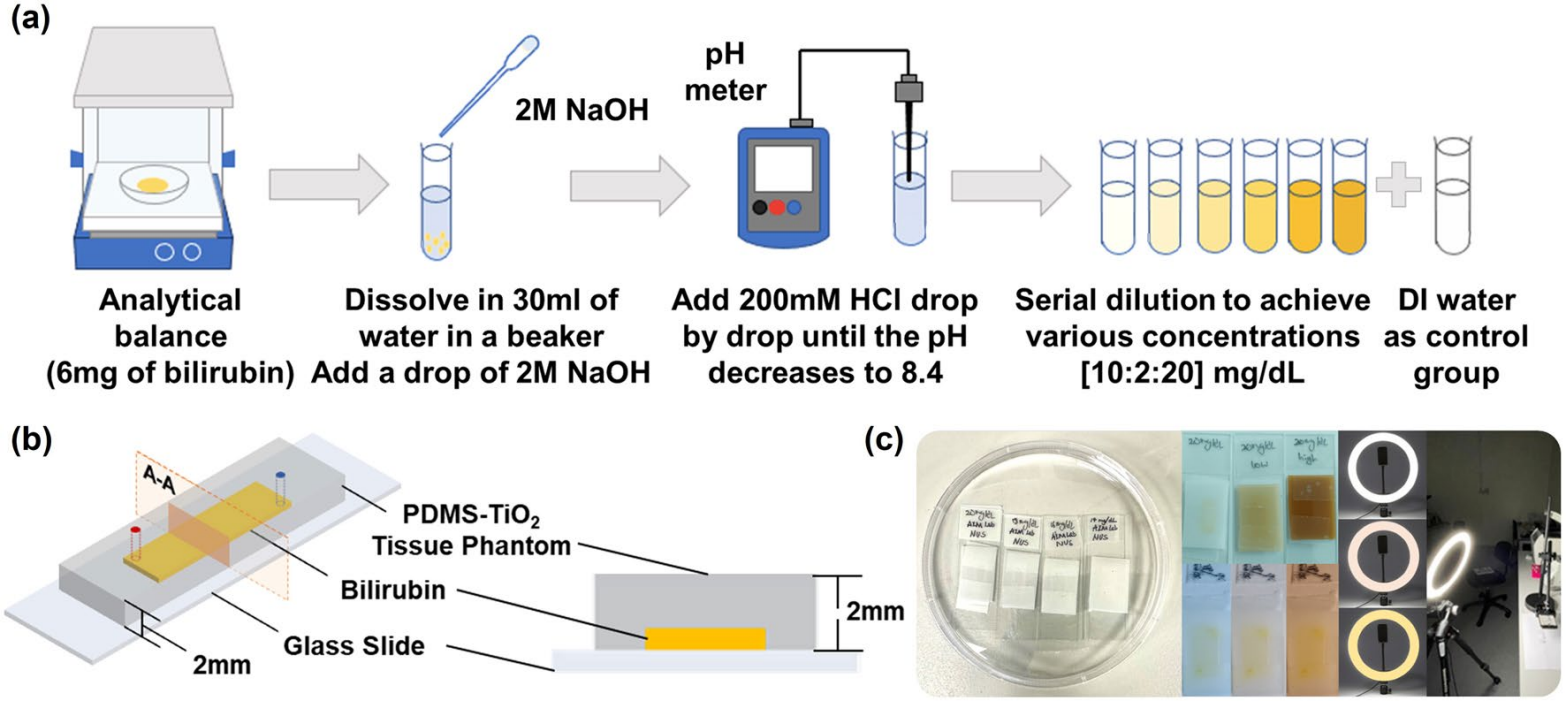
Overall PDMS tissue phantom image collection overview. (**a**) Preparation procedures of bilirubin solutions with different concentrations via serial dilution. (**b**) PDMS-TiO_2_ tissue phantom design to mimic the bilirubin deposition on the skin tissue in humans. (**c**) Fabricated PDMS-TiO_2_ tissue phantom samples and corresponding PDMS-TiO_2_ tissue phantom image collection environment set-up.

As shown in Fig. 6a, 6 mg bilirubin powder was measured with an analytical weighing balance and transferred to a glass beaker with 30 ml of deionized (DI) water. A drop (approximately 20 µl) of 2 M NaOH solution was added to dissolve the bilirubin powder completely after which 200 mM HCl solution was slowly titrated until a pH of 8.4 was achieved in the bilirubin solution. Serial dilution with DI water was then performed to obtain bilirubin solutions with concentrations of 10, 12, 14, 16, 18, and 20 mg/dl. Together with DI water (0 mg/dl) as a control group, a total of seven concentrations were prepared for PDMS tissue phantom in vitro experiments. 100 μl of each solution was loaded into a 96-well microplate for spectrophotometer scanning.

Microfluidic channels were fabricated by standard soft lithography as shown in Fig. 6b. TiO_2_ was used as a scattering agent in the preparation of a 2 mm thick PDMS-TiO_2_ tissue phantom layer, replicating the optical properties of human tissues^32^. A 10:1:0.01 ratio by mass of silicon base to curing agent to TiO_2_ was utilized. The mixture was then poured onto a silicon mould, followed by degassing for at least 1 h 30 min and baking at 70 °C for 2 h. After cutting out the channels, the film tissue phantom was then bonded to standard microscope glass slides by oxygen plasma treatment. Inlet and outlet microfluidic ports were punched to allow bilirubin solutions of various concentrations (10 to 20 mg/dl) to be loaded into the channel. To represent the accumulation of bilirubin deposition at different sites of the skin, similar preparation procedures were conducted to prepare PDMS-TiO_2_ tissue phantoms with a thickness of 1 mm, 2 mm, and 3 mm respectively. The same ratio by mass of silicon base to curing agent to TiO_2_ but 18 g and 54 g mixture of PDMS-TiO_2_ were used respectively. Different ratios by mass (10:1:0.015 and 10:1:0.02) of silicon base to curing agent to TiO_2_ were used to prepare tissue phantoms with different scattering properties^46^.

### Data acquisition and image pre-processing

#### Image collection protocol and parameters setting

The image collection was conducted in a controlled lab environment. An Oppo Reno 5Z mobile phone main camera (48MP sensor, aperture F1.7) was used to collect images throughout the experiments as shown in Fig. 6c. An A4-sized sheet of standard white copy paper was pasted on a flat surface as a background for the samples. Two camera modes (auto and manual) were utilized in the image collection process. Depending on the specific experimental condition, camera parameters such as ISO and WB were specifically adjusted. A summary of the various tissue phantom and image collection parameters used for each parametric study is provided in Supplementary Table 1.

#### White balance (WB) correction

**Figure 7:**
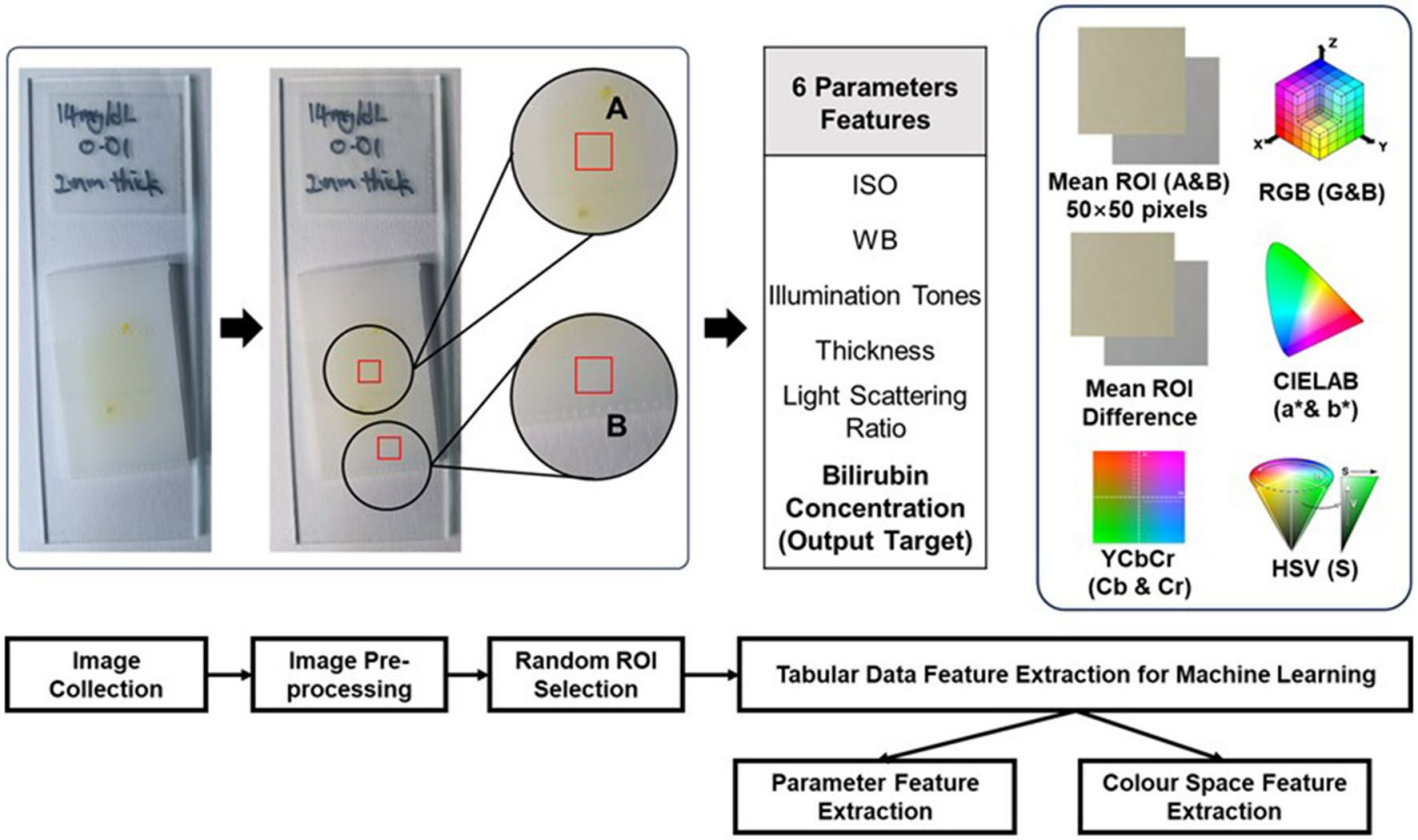
Graphical summary of data extraction process. (Left to right) Images are first obtained using a mobile phone camera and WB-corrected. Two regions of interest (ROI)—(**A**) bilirubin-containing and (**B**) bilirubin-free regions—are then isolated from the images of the tissue phantom. From these, 11 colour space features are derived and tabulated with 6 parameter features and used as inputs for the ML models.

The collected images were undergone a few image pre-processing steps, such as WB correction and colour space conversion (Fig. 7). WB correction refers to the process of adjusting the colour cast of digital images to achieve a neutral colour rendering. In this study, we applied different colour constancy algorithms (GW, MSGP, MaxRGB, and CH) to reduce the effects of different light sources on images, generating a machine learning dataset with less noise. The ground truth image was determined by the balanced light temperature (5600 K) and was compared with WB-corrected images based on the Eq. (1) to calculate the angular error.

1 $${err}_{angular}={{\text{cos}}}^{-1}\frac{\left({\rho }^{E}\cdot {\rho }^{Est}\right)}{\Vert {\rho }^{E}\Vert \Vert {\rho }^{Est}\Vert }$$

where ${\rho }^{E}$ represents the RGB of actual illumination in the ground truth images, ${\rho }^{Est}$ represents the RGB of the corrected illuminations based on different white balance algorithms. ‘.’ represents the vector dot product. A small angular error would indicate better correction performance of the WB correction algorithms on tissue phantom RGB images as compared to the ground truth image^47–49^.

#### Colour spaces conversion and feature extraction

For each WB-corrected image, a square ROI free of glare and shadow with a fixed number of pixels (50 × 50 pixels) was selected (Fig. 7). The mean pixel intensities in the R, G and B channels were then extracted respectively, obtaining a set of mean RGB pixel values^50^. The WB-corrected ROI was also mapped onto different colour spaces (CMY(K), L*a*b*, HSV, YCbCr and L*u*v). The mean values obtained from different colour spaces of the same ROI were used to evaluate the correlation and potential as image features. The representative formulae of CMY(K) in Eq. (2), L*a*b* in Eq. (3), HSV in Eq. (4), YCbCr in Eq. (5) and L*u*v* in Eq. (6) are shown below^38,51–53^:

CMYK:

$${C}{\prime} = 1 - R / 255; {M}{\prime} = 1 - G / 255; {Y}{\prime} = 1 - B / 255;$$

$$K = min(C, min(M, Y));$$

$$C = ({C}{\prime} - K) / (1 - K);$$

$$M = ({M}{\prime} - K) / (1 - K);$$

2 $$Y = ({Y}{\prime} - K) / (1 - K);$$

L*a*b*:

$${C}^{*}={\left({\left({a}^{*}\right)}^{2}+ {\left({b}^{*}\right)}^{2}\right)}^\frac{1}{2};$$

$${h}^{\diamond }=arctan\frac{b*}{a*};$$

3 $$\Delta {{E}^{*}}_{ab}={\left({\left({\Delta L}^{*}\right)}^{2}+ {\left(\Delta {a}^{*}\right)}^{2}+ {\left(\Delta {b}^{*}\right)}^{2}\right)}^\frac{1}{2},$$

where ${C}^{*}$ indicates chroma, ${h}^{*}$ indicates hue $\Delta {{E}^{*}}_{ab}$ indicates the composite colour difference for the changes of L*, a*, and b* components.

HSV:

$$\left\{\begin{array}{l}max=max( R, G,B )\\ min = min(R, G ,B)\end{array}\right. ;$$

$$H=\left\{\begin{array}{l}0^\circ , if max=min\\ 60^\circ \times \frac{G-B}{max-min}, if max=R and G\ge B\\ 60^\circ \times \frac{G-B}{max-min}+360^\circ , if max=R and G\ge B\\ 60^\circ \times \frac{B-R}{max-min}+120^\circ , if max=G\\ 60^\circ \times \frac{R-G}{max-min}+240^\circ , if max=B\end{array} ;\right.$$

4 $$S=\left\{\begin{array}{l}0, if max=0\\ 1- \frac{min}{max}, otherwise\end{array} ;\right. V=max ;$$

YC_*b*_C_*r*_:

$$\left\{\begin{array}{l}{Y}^{{{\prime}}} = 0.299{R}^{{{\prime}}}+ 0.587{G}^{{{\prime}}}+0.114{B}^{{{\prime}}}\\ {P}_{b} = ({B}^{{{\prime}}} - {Y}^{{{\prime}}})/{k}_{b}= -0.1687{R}^{{{\prime}}}- 0.3313{G}^{{{\prime}}}+0.500{B}^{{{\prime}}}\\ {P}_{r} = ({R}^{{{\prime}}} -{Y}^{{{\prime}}})/{k}_{r} = 0.500{R}^{{{\prime}}}- 0.4187{G}^{{{\prime}}}-0.0813{B}^{{{\prime}}}\end{array}\right. ;$$

5 $$\left\{\begin{array}{l}Y = {219 * Y}^{\mathrm{^{\prime}}} +16\\ {C}_{b} = {224 * P}_{b} +128\\ {C}_{r} = 224{* P}_{r} +128\end{array}\right. ;$$

L*u*v*:

$${L}^{*}=\left\{\begin{array}{l}{\left(\frac{29}{3}\right)}^{3}Y/{Y}_{n}, Y/{Y}_{n}\le {\left(\frac{6}{29}\right)}^{3}\\ 116{(Y/{Y}_{n})}^{1/3}-16, Y/{Y}_{n}>{\left(\frac{6}{29}\right)}^{3}\end{array}\right. ;$$

6 $${u}^{*} =13{L}^{*} \cdot ({u}^{\mathrm{^{\prime}}} - {u}_{n}^{\mathrm{^{\prime}}}) ; {v}^{*} = {13L}^{*}\cdot ({v}^{\mathrm{^{\prime}}} - {v}_{n}^{\mathrm{^{\prime}}}) ;$$

### Machine learning algorithms

This study tested several well-established supervised ML models (DT, KNN, SVM, RF and LightGBM) to analyse the dataset collected from the tissue phantom images with different bilirubin concentrations. Decision tree (DT) is a non-parametric supervised learning model for classification and regression. The complexity and fitness of the model are determined by the number of depth layers. A DT is able to handle both numerical and categorical data. Each layer represents a feature that categorizes the input unknown data into a specific class or range in classification and regression respectively. K-Nearest Neighbour (KNN) is a supervised machine learning algorithm that relies on the similarities and proximity between the labelled known data and the unknown new data for prediction. It calculates the distance between the input data and its corresponding K number of neighbouring known data, returning the mode and the mean of the K labels in classification and regression respectively. The core idea of Support Vector Machine (SVM) is to minimize the structural risk and empirical risk of the model by constructing a hyperplane (i.e., regression function) within a given tolerance error range. SVM is robust to noise in the training data and it can address both linear and non-linear relationship in the case for solution. Commonly used kernel functions include linear kernel, polynomial kernel, radial basis function kernel (RBF), etc. Random forest (RF) is an effective ensemble learning algorithm that achieves accurate prediction by fusing multiple DTs. During the training phase, bootstrap sampling and random feature selection strategies are employed to bring diversity to the model, thereby building a series of independent DTs. In regression tasks, a RF combines the prediction results of individual DTs and usually use the average as the final prediction value to achieve stable and accurate prediction performance. In this study, the optimized RF model is applied to process the colour features of the sample to be predicted to obtain the predicted value of bilirubin concentration. Light Gradient Boosting Machine (LightGBM) is also an effective ML algorithm based on gradient boosting trees. It is suitable for solving both regression and classification problems. Its core principle is to combine multiple weak learners (usually DTs) to gradually optimize the fitting effect of the model through the forward distribution algorithm. In each iteration, the LightGBM model learns a new weak learner to fit the residuals of the previous model, thereby gradually improving the accuracy of the prediction.

Models were run using the present libraries in Python. A total of 3465 images in the dataset were divided into 2772 (80%) training set images and 693 (20%) test set images. The images in both sets did not mix or overlap. Hyperparameter tuning was also conducted for each model to ensure that the dataset is well-fitted into the model, thereby optimizing the accuracy and robustness of the model's predictions. The optimal hyperparameters were derived using cross-validated grid-search by GridSearchCV.

In the classification task, the final output estimated whether the concentration of the bilirubin in each image passed a threshold (15 mg/dl), which indicates the status of normal/jaundice. In the regression task, the final output estimated the bilirubin concentration from each test image of the PDMS-TiO_2_ tissue phantom.

### Data analysis

Linear regression was performed to derive the correlation between the bilirubin concentration and the RGB pixel value, estimating the effect of confounders on image analysis and the characteristics of the colour space features. The accuracy and sensitivity of the linear regression were determined by the R^2^ value and the gradient of the graph respectively. In this study, supervised ML models (i.e., KNN, Decision Tree, Random Forest, SVM, LightGBM, etc.) were utilized for the image analyses. The dataset used for training and testing comprised the tabular data from the WB-corrected PDMS-TiO_2_ tissue phantom images with 17 feature labels, including features from biological and external environmental confounding factors, as well as the colour space information with the highest correlation to bilirubin concentration such as RGB, the S channel from the HSV colour space, the a* and b* channel from the L*a*b* colour space, as well as the u* and v* channel from L*u*v* colour space. The features also include the RGB differences between ROIs with and without bilirubin content. A single-label binary classification task (normal and jaundice) and a bilirubin level (10 to 20 mg/dl) prediction regression task were performed with the different models. Model performances were compared by different metrics. In the classification task, a confusion matrix, which includes the accuracy, precision, sensitivity, and specificity of classification, was calculated based on the true negative (TN), true positive (TP), false negative (FN), and false positive (TP) predictions in Eqs. (7) to (10):

7 $$Accuracy= \frac{TP+TN}{TP+TN+FP+FN}$$

8 $$Precision= \frac{TP}{TP+FP}$$

9 $$Sensitivity= \frac{TP}{TP+FN}$$

10 $$Specificity= \frac{TN}{TN+FP}$$

The ROC curve and corresponding AUC score was also evaluated to describe the ability of different models in the classification task^54^.

The regression models were trained and tested using two separate datasets, one with 6 features and the other with 17 features. The performances of each model with 6 features and 17 features were compared to identify if there was an improvement in the model performance when including more features. The R^2^ value and MSE were calculated to quantitatively evaluate the performance of each regression model in bilirubin level determination and to evaluate the important features in each model respectively. To compare the performance of the models, a 10-fold cross-validation was performed 10 times, resulting in 100 accuracy results for each model. By randomly permutating the training variables in each model, the changes in MSE were compared. The importance of each feature was then determined based on the changes in MSE^40^.

### Statistical analysis

All statistical analyses were performed in OriginPro 2021b software. A significance level of 95% was utilised as the significance threshold throughout the study. An F-test was conducted to determine the significance of the correlation between pixel value and various bilirubin concentrations in each linear regression model. A regression slope t-test and Student’s t-test were conducted for every regression plot to evaluate the significance of the confounding factors on the colour space information. Non-parametric Kruskal–Wallis H test was used to evaluate the effectiveness of different WB correction algorithms and the performance of the various machine learning models. A post-hoc Dunn’s test was followed to conduct a pairwise comparison between models. A non-parametric Wilcoxon signed-rank test was also performed to compare the machine learning model performance before and after the modifications made in data sets and data features (i.e., raw vs. WB-corrected; 6 features vs. 17 features; inclusion vs. exclusion of features from different colour spaces; inclusion vs. exclusion of ROI as a baseline).

### Supplementary Information

Supplementary Information.

## Data Availability

The datasets generated and /or analysed during the current study are available from the corresponding author upon reasonable request and permission.
