# Supplementary material for: Application of machine learning algorithms for accurate determination of bilirubin level on in vitro engineered tissue phantom images
Source: Sci Rep. 2024 Mar 12;14:5952. doi: 10.1038/s41598-024-56319-4 (PMC10928098; doi:10.1038/s41598-024-56319-4)
Supplement: Supplementary file 1 — Supplementary Information. [file 41598_2024_56319_MOESM1_ESM.docx]

**Supplementary Information**

**Application of Machine Learning Algorithms for Accurate Determination of Bilirubin Level on *In Vitro* Engineered Tissue Phantom Images**

**Yijia Yuan^1, 2^, Jiayao Huang^1^, Jiachen Yu^1, 3^, Justin Kok Soon Tan^1, 2^, Kevin Ziyang Chng^4^, Jiun Lee^5, 6^, Sangho Kim^1, 2,^ ***

^1^Advanced Innovation in Micro/Nanoengineering (AIM) Laboratory, Department of Biomedical Engineering, National University of Singapore, 119276, Singapore
^2^N.1 Institute for Health, National University of Singapore, 119276, Singapore

^3^College of Biomedical Engineering and Instrument, Zhejiang University, 310027, China

^4^AI Singapore, 117602, Singapore

^5^Department of Neonatology, National University Health System, 119228, Singapore

^6^Department of Paediatrics, National University of Singapore, 119228, Singapore

***Corresponding author**

Sangho Kim, PhD

Department of Biomedical Engineering, National University of Singapore

9 Engineering Drive 1, Singapore 117575, Block E2 #03-20

Singapore 117583

Phone: 65-6516 6713

Fax: 65-6872 3069

Email: [bieks@nus.edu.sg](mailto:bieks@nus.edu.sg)

**Supplementary Table 1:** Summary of the Samples and the Parameter Settings

| Figures  Sample Type | ISO | WB | Light Scattering Ratio | Thickness | Illumination Tone | Light Intensity |
| --- | --- | --- | --- | --- | --- | --- |
| Figure 1  Bilirubin Solutions | N. A | | | | | |
| Figure 2a  Tissue Phantom Images | 500 | 5000K | 0.010 | 1, 2 & 3 mm | White | High |
| Figure 2b  Tissue Phantom Images | 500 | 5000K | 0.010, 0.015 & 0.020 | 2mm | White | High |
| Figure 2c  Tissue Phantom Images | 500 | 2000K, 5000K, & 8000K | 0.010 | 2mm | White | High |
| Figure 2d  Tissue Phantom Images | 100, 500 & 1000 | 5000K | 0.010 | 2mm | White | High |
| Figure 2e  Tissue Phantom Images | 500 | 5000K | 0.010 | 2mm | White, Off-white & Yellow | High |
| Figure 2f  Tissue Phantom Images | 500 | 5000K | 0.010 | 2mm | White | Low, Medium (Mid) & High |
| Figure 3  Tissue Phantom Images | 500 | 5000K | 0.010 | 2mm | White | High |
| Figure 4  Tabular Data (Tissue Phantom Image Features) | 100-1000 | 2000K-8000K | 0.010, 0.015 & 0.020 | 1, 2 & 3 mm | White, Off-white & Yellow | Low, Medium (Mid) & High |
| Figure 5  Tabular Data (Tissue Phantom Image Features) | 100-1000 | 2000K-8000K | 0.010, 0.015 & 0.020 | 1, 2 & 3 mm | White, Off-white & Yellow | Low, Medium (Mid) & High |

**Supplementary Figure 1**: Spectral Characterization of Different Illumination Tones. Different illumination tones (white, off-white and yellow) were tested, white illumination has demonstrated the highest peak at 449nm, while the off-white illumination and yellow illumination peak at 451nm and 457nm respectively.

**Supplementary Figure 2:** Scatter plot of the pixel value of bilirubin concentrations in PDMS-TiO_2_ tissue phantom samples with different image capture distances. No statistically significant difference was observed in images with varying capture distances (P > 0.05).
